# Diagnostic errors in fatal medical malpractice cases in Shanghai, China: 1990–2015

**DOI:** 10.1186/s13000-019-0785-5

**Published:** 2019-01-31

**Authors:** Pan Gao, Xiaoqiang Li, Ziqin Zhao, Nong Zhang, Kaijun Ma, Liliang Li

**Affiliations:** 1 Department of Pathology, School of Basic Medical Sciences, Fudan University, Shanghai, 200032 People’s Republic of China; 2 Shanghai Diabetes Institute, Department of Endocrinology and Metabolism, Shanghai Jiao Tong University Affiliated Sixth People’s Hospital, Shanghai, 200032 People’s Republic of China; 3 Department of Epidemiology, School of Public Health, Fudan University, Shanghai, 200032 People’s Republic of China; 4 Department of Forensic Medicine, School of Basic Medical Sciences, Fudan University, 138 Yixueyuan Road, Xuhui District, Shanghai, 200032 People’s Republic of China; 5 Shanghai Key Laboratory of Crime Scene Evidence, Shanghai Public Security Bureau, 803 North Zhongshan Road, Hongkou District, Shanghai, 200083 People’s Republic of China

**Keywords:** Diagnostic errors, Medical disputes, China, Risk factors

## Abstract

**Background:**

Medical disputes remain unabated in China. Previous studies have shown the changes of diagnostic discrepancy over time in developed countries, but diagnostic discrepancy remains understudied in China, especially in the setting of medical disputes. We sought to describe the year-based changes of diagnostic discrepancies in medical disputes, and to identify factors associated with classes of diagnostic discrepancy.

**Methods:**

We conducted a retrospective cohort study of all medically disputed cases from 1990 through 2015 in Shanghai, China, with use of necropsy as the gold standard for diagnosis. Cases were grouped based on national legislative eras. Diagnostic discrepancy was classified as major errors (class I and II), minor errors (class III and IV), no discrepancy (class V) and undetermined (class VI) based on discrepancy severity.

**Results:**

There were 482 medical disputes. Cases were predominantly males (male: female = 1.6:1) and concentrated in patients less than 10 years old or between 50 and 70 years. Major and minor discrepancy accounted for 51.7 and 34.8%, respectively. Fifty-five cases (11.2%) were non-discrepant (Class V). The dispute rate remained high before the first round of legislation (mean 0.31 per 1 million patients) but declined dramatically afterwards (R^2^ = − 0.82, *p* < 0.001 for time trends). Over the national legislative eras, the annual number of cases with diagnostic errors declined steadily. Incidence rates of discrepancy decreased significantly for class I (R^2^ = − 0.73, *p* = 0.024), II (R^2^ = − 0.48, *p* = 0.013), III (R^2^ = − 0.69, *p* < 0.0001), IV (R^2^ = − 0.69, p < 0.0001) and V discrepancy (R^2^ = − 0.58, *p* = 0.0018). Diseases from the respiratory system had significantly lower risks of any diagnostic errors (OR = 0.48, 95% 0.24–0.95, *p* = 0.036). A neoplasm carrier increased by 92% the risk of any diagnostic error (OR = 1.92; 95%CI 1.18–3.14; *p* = 0.009) and hypertension reduced by 78% the risk of minor errors (OR = 0.22, 95%CI 0.06–0.91, *p* = 0.036). Severity of discrepancy relieved over years and associated with ageing in patients with cardiovascular diseases (*p* = 0.01).

**Conclusions:**

The rate of fatal medical disputes and diagnostic discrepancy declined after stepwise legislations in China. Respiratory diseases, neoplasm carrier and hypertension could be independent predictors for assessing diagnostic errors.

## Background

Medical disputes can present an unwelcome emotional and economic burden in the world, including China. A recent study from the United States has found that 7.4% of all physicians confronted with a medical dispute annually, with 1.6% having a claim leading to a mean payment of $274,887 [1]. One insurance company from Germany reported that about 4500 out of 108,000 (4.2%) insured doctors were involved in medical disputes each year, with settlement of cases in 30, and 10% going to a civil court [2]. Though it is prohibitively difficult to obtain an exact data in China, it was estimated that at least 420,000 patients may die each year (approximate 1150 patients per day) from preventable medical errors [3], and approximately one RMB in malpractice compensation might be an additional indirect cost leading to hospital losses of 6.7 times or more [4].

Diagnostic errors underlie largely the great burden from medical disputes. Pathologists from the United Kingdom Royal College have reported that the rate of clinical diagnostic errors reached 75% [5]. The misdiagnosis rate was up to 36.24% in Zhejiang province, China from 1950 through 1999 [6]. A recent study by us concluded that 35.9% of dispute claims were incorrectly diagnosed from 2004 to 2013 [3].

The scenario of China’s diagnostic levels remains obscure, especially in the setting of medical disputes. Currently, there are few literatures reporting diagnostic errors that were only accessible in Chinese language. Each of these earlier reports has limitations, including use of older data with limited coverage of different medical settings and specialties, the presentation of only descriptive data without identifying potential risk factors for predicting diagnostic discrepancies, and a lack of analysis of time trends of medical dispute incidence.

The present study sought to retrospectively review all fatal medical dispute cases in the biggest metropolitan area Shanghai, China, to provide the time trends of medical disputes and diagnostic discrepancies over a lengthy period 1990–2015. Diagnostic discrepancy was classified for each case based on discrepancy severity with use of autopsy as the gold standard for diagnosis. We aimed to identify factors associated with the different classes of diagnostic discrepancy. This study represents the first one to provide multifaceted look at China’s medical disputes and provide potential predictors for preventing diagnostic discrepancies.

## Methods

### Settings

There is no uniform registry system currently available in China regarding medical disputes, making it difficult to obtain a nationwide data. Shanghai is the biggest metropolitan area in China which is located in Eastern China with its residents over 30 million (about 2.5% of China’s overall population). Shanghai ranks as one of the top cities which provide the highest medical services in China. Medical units serve both permanent residents in Shanghai and also large population from outside of the city with the average outpatients of 0.12 billion per year (approximate 1/13 of China’s population) during the present studied period. According to China law, a medical dispute is primarily addressed by the Medical Association which is affiliated to the local Bureau of Health. When a fatal medical dispute is claimed to the Medical Association in Shanghai, a systemic autopsy will be ordered and the disputed case will be normally referred to the Department of Pathology, School of Basic Medical Sciences, Fudan University for autopsy examination.

The certified department is responsible for conducting a complete autopsy examination, making a clear pathological diagnosis and certifying the cause of death pertaining to each referred fatal case. The autopsy procedures/protocols at the department have been consistent for the past decades. Before autopsy, a staff pathologist in the University Department initially collected the detailed clinical information. Two attendant pathologists then conducted the macroscopic and microscopic examinations. Toxicology or microbiology detection was issued when necessary. Every two weeks the results of necropsies were presented and discussed in the clinical pathology conferences. The main diagnoses were listed according to the severity of abnormality and were approved by a three-tiered review board before the autopsy report was released. Each body was kept in freezing condition until autopsy. The role of the University Department, as the referral center of medical disputes in Shanghai remained unchanged over the last decades.

### Study design

This study was based on all medical malpractice cases investigated at the abovementioned department during the period from 1990 through 2015. The spanned years were subgrouped as 1990–1994, 1995–1999, 2000–2004, 2005–2010, and 2011–2015. Case inclusion criteria for participation in the study were: (1) malpractice cases were closed and ascertained by authorities; (2) clinical information was obtained; (3) patient’s age was over 28 days old; (4) case was fatal and underwent a complete autopsy with examination of all organs. Stillbirth cases were excluded because these cases had indeterminate death time. Newborn and fetus cases were also excluded since some cases were missing of clinical data, or the evaluation of diagnostic discrepancy was complicated by their mothers’ health state. Any case with its pathological diagnosis uncertain was excluded.

For each case, its clinical and demographic information were extracted from the clinical documents. These information included patient’s age, gender, clinical complaint on the last admission, involved medical units and specialty, patient’s medical history, date on admission, admission record, treatment procedures and death certificate. The main pathological diagnoses and cause of death were extracted from autopsy report. The length of hospital stay was calculated based on clinical documents (length of hospital stay = death date-admission date). Patient’s complaint was compared with the pathological major diagnoses to determine whether patient’s complaint on admission matched or not with the final cause of death. An indicative complaint was assigned if patients’ subjectively reported symptoms matched objectively diagnosed cause of death, otherwise a non-indicative complaint was assigned. Patients’ past history of any cardiovascular risk factors (i.e. hypertension, diabetes mellitus), carrying any type of tumor (benign or malignant) or history of surgery were recorded from clinical data or by patients’ family members. For deaths related to surgical resection, it was checked whether they died during perioperative period. Perioperative period defines as preoperative period (ward admission, approximate 5–7 days before surgical procedure), intraoperative period (anesthesia and surgery) and postoperative periods (recovery for about 7–12 days in ward). This study was retrospective without physical review and re-examination of the toxicology, serology or microscopy.

### Classification of diagnostic discrepancy

Before classification of diagnostic discrepancy, each of clinical and pathological diagnoses was classified as major (primary cause of death and principle underlying contributors) plus minor diagnosis (antecedent disorders or other important conditions) as described previously by us and other researchers [3, 7–9]. The major and minor diagnoses were thereafter compared between clinical and pathological documents to determine whether the clinicopathological diagnoses had major discrepancy, minor discrepancy, non-discrepancy or were non-classifiable. A 6-level categorization of diagnostic discrepancy (Class I, II, III, IV, V and VI) was then employed in this study. Major discrepancy (Class I and II), minor discrepancy (Class III and IV), non-discrepancy (Class V) and non-classifiable discrepancy (Class VI) were defined in reference to previous categorizations [7, 8, 10] with minor modifications (Table 1). Cases with diagnostic errors were accordingly denoted as those with major or minor diagnostic discrepancy. In other words, cases with major diagnostic errors corresponded to those with major clinicopathological discrepancy (Class I and II discrepancy) and cases with minor diagnostic errors corresponded to those with minor clinicopathological discrepancy (Class III and IV discrepancy). Cases with any diagnostic error represented those with either major or minor discrepancy (Class I to IV discrepancy), while cases without any error corresponded to those with non-discrepancy (Class V).

**Table 1:** Classification system of diagnostic discrepancy according to previous categorizations [7, 8, 10] with minor modifications

Classifications	
Major discrepancies-*Class I*	
Knowledge of major diagnosis would have prolonged survival or cured patients without leading to deaths (e.g. Patients with acute pancreatitis were treated as myocardial infarction)	
Major discrepancies-*Class II*	
Knowledge of major diagnosis would not have changed survival even with correct diagnosis (e.g. Patients with bone fractures developed pulmonary emboli)	
Minor discrepancies-*Class III*	
Discrepant minor diagnosis was not directly related to cause of death, but would affect the prognosis if not treated (e.g. Disseminated neoplastic patients with pneumonia)	
Minor discrepancies-*Class IV*	
Clinically non-diagnosed occult diseases that would not affect the prognosis but may have epidemiological or genetic importance (e.g. Symptomless liver steatosis, prostatomegaly)	
Non-discrepancy- *Class V*	
Non-discrepant diagnoses	
Non-classifiable cases- *Class VI*	
Patients died immediately after admission without any diagnostic procedures, or the clinical diagnoses were blank, missing, or ambiguous	

A single class of discrepancy was assigned to each case. In the event of more than one major diagnoses, the most severe was chosen. In this event, determination of severer diagnosis relies on the clinical course and pathological examination. For example, a patient was diagnosed with hepatocellular carcinoma. He died after 2-month stay at hospital and the pathological diagnosis was carcinoma complicating pneumonia. Obviously, pneumonia was the most severe diagnosis (major diagnosis) and the cause of death in pathology. This case would be classified as major discrepancy since clinicians neglected the severe pneumonia developed after long bedridden time. In contrast, if a patient with invasive cancer died soon after hospitalization and autopsy revealed focal or mild pneumonia, the major cause of death would be progressive cancer and pneumonia would be a minor diagnosis. In this event, the discrepancy would be minor because invasive cancer would be the most possible cause for his death. The present study only focused on diagnostic errors. Therapeutic, surgical or care errors were not in the scope of current study, though they could also lead to medical malpractice. Diagnostic discrepancies were classified after agreement of three pathologists. In a few cases that received no consistent conclusion, a senior pathologist uninvolved in the study was consulted.

### Classification of medical setting, specialty and organ system

In urban areas of China, a three-tiered system of medical units is used to classify public hospitals which includes community health care centers (primary level), district hospitals (secondary level) and city hospitals (tertiary level) [3]. But in reality, classification of hospitals cannot be simply based on area distribution. For example, some district hospitals are affiliated to universities (university hospitals) and hence gain more medical resources. These medical units are actually tertiary. In view of the confusion, the Ministry of Health in China has made comprehensive assessment of each public medical unit to classify medical units and made the classification results open and accessible to the public. This study adopted the classification system and classified all involved medical units as tertiary, secondary and primary ones, where tertiary units include both university hospitals and non-affiliated city-level hospitals. Secondary units are district hospitals except those affiliated to universities, and primary units cover community hospitals and primate clinics.

Specialty defines as the hospital ward where patients were hospitalized at their latest admission. If patients were hospitalized to more than one ward in the latest admission or admitted for multiple times previously, we assessed and classified the specialty up to the one at which patients died. Hospital wards were classified as internal department, surgery department, emergency room, and other wards such as department of psychiatry, department of eyes, ear, nose and throat (EENT), department of dermatology and department of traditional Chinese medicine.

Diseases were classified according to organ system. Categorization of organ systems included circulatory, respiratory, digestive, central nervous, urogenital, endocrinal and metabolic, and EENT systems.

### Statistical analysis

Cases were calculated as numbers in total with percentages when necessary. The Cochran-Mantel-Haenszel χ2 tests were used to assess the differences among categorical variables. The time trends in medically disputed cases were assessed using χ2 tests and Poison regression models. Incidence rates of medical disputes and diagnostic discrepancies were calculated based on patients’ hospitalization data released by the Health Authority, Shanghai, China. Multivariate logistic regression analysis was then performed to examine the factors associated with any discrepancy (class I to VI), major discrepancy (Class I and II) and minor discrepancy (Class III and VI). Tests yielding 2-tailed values of *p* ≤ 0.05 was considered statistically significant. All analyses were conducted using SAS version 9.2 (SAS Institute, Inc. Cary, NC).

## Results

### Epidemic and demographic features of medical disputes from 1990 to 2015

From 1990 to 2015, there were 3186.51 million patients hospitalized in Shanghai. A total of 649 cases (1 patients per 5 million hospitalization) that were fatal medical disputes were reported during the studied period. Among the 649 cases, 155 were stillbirth or died within 28 days after birth. Another 12 cases had incomplete clinical demographic data. Hence, the left 482 cases (1.5 per 10 million hospitalizations) met our inclusion criteria. Distribution of these medical disputes (*n* = 482) by patients’ age, gender, medical setting, specialty and organ systems were summarized in Table 2.

**Table 2:** Distribution of medical disputes and ascertained diagnostic errors (class I-IV) by age, gender, medical settings, specialty and involved organ system

Characteristics	No. disputes	No. diagnostic errors	Percent of errors (95% CI)
Age, years			
(0, 10)	46	33	71.7 (58.7–84.8)
[10, 20)	22	13	59.1 (38.6–79.6)
[20, 30)	41	35	85.4 (74.6–96.2)
[30, 40)	53	46	86.8 (77.7–95.9)
[40, 50)	71	65	91.6 (85.1–98.0)
[50, 60)	84	73	86.9 (79.7–94.1)
[60, 70)	79	73	92.4 (86.6–98.3)
[70, 80)	62	56	90.3 (82.9–97.7)
≥ 80	24	22	91.7 (80.6–100.0)
Gender			
Female	186	162	87.1 (82.3–91.9)
Male	296	254	85.8 (81.8–89.8)
Medical Settings			
Tertiary	298	251	84.2 (80.1–88.4)
Secondary	157	141	89.8 (85.1–94.5)
Primary	27	24	88.9 (70.8–97.7)
Specialty			
Internal	172	147	85.5 (80.2–90.7)
Surgery	144	127	88.2 (82.9–93.5)
Emergency	119	103	86.6 (80.4–92.7)
Pediatrics	16	12	75.0 (53.8–96.2)
Obstetrics	14	13	92.9 (79.4–100.0)
Other wards^a^	17	14	82.4 (64.2–100.0)
Organ systems			
Circulatory	151	132	87.4 (82.1–92.7)
Respiratory	166	141	84.9 (79.5–90.4)
Digestive	104	94	90.4 (84.7–96.1)
Central nervous	37	28	75.7 (61.9–89.5)
Urogenital	14	13	92.9 (79.4–100.0)
Other systems^b^	10	8	80.0 (55.2–100.0)

^a^includes departments of psychiatry (*n* = 8), eyes, ear, nose and throat (EENT, *n* = 7), dermatology (*n* = 1), traditional Chinese medicine (*n* = 1)

^b^includes endocrinal and metabolic system (*n* = 6) and EENT system (*n* = 4)

**Fig. 1:**
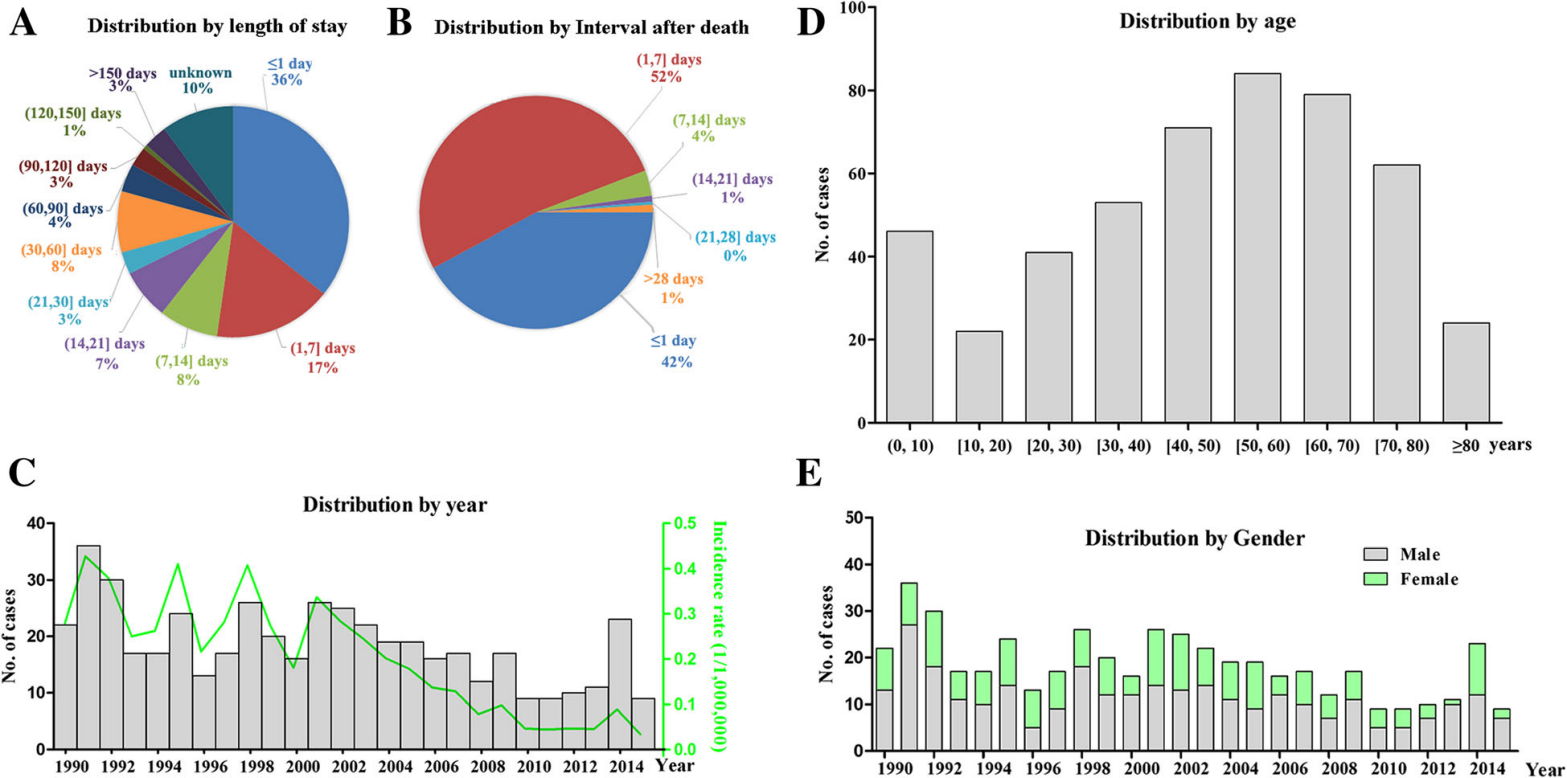
Distribution of medical malpractice cases were shown. **a** Distribution of medical malpractice cases by patients’ length of latest hospital stay. **b** Distribution of medical malpractice cases by postmortem time interval after deaths. **c**-**e** Distribution of medical malpractice cases by year period, patients’ age and gender. The incidence rate of medical malpractice in each year was also plotted (**c**). Chi-square tests showed the incidence rate was significantly decreasing over the 26-year spanning (correlation efficiency (R^2^) = − 0.862, *p* < 0.001 for time trends)

All the 482 cases were examined within an average of 3.0 days (± 6.6 days) after death (median 2 days). Forty-two percent (42%) were autopsied within 1 day and 52% were between 1 and 7 days after notification of death (Fig. 1a). In total, 433 cases (89.8%) had a known length of hospital stay with the mean duration as 28.0 ± 71.5 days (median 4 days). Over one-third of cases had been staying in hospital for less than 1 day and another 17% were within 7 days (Fig. 1b).

The number of medical disputes decreased significantly over years from 22 cases in 1990 to 9 cases in 2015, making a 59.1% decrease. Accordingly, the annual dispute rate was 0.26 per million hospitalizations in 1990 but declined dramatically afterwards to only 0.034 per million hospitalizations in 2015 (Fig. 1c, correlation efficiency (R^2^) = − 0.862, *p* < 0.001 for time trends). Cases were concentrated in patients less than 10 years and between 50 and 70 years old (Fig. 1d) and predominantly males (male: female ratio = 1.6:1) (Fig. 1e).

### Diagnostic discrepancy of the medical disputes

A total of 471 cases (97.7%) had different extents of clinicopathological diagnostic discrepancy (Class I, II, III, IV and V). Of these cases, 51.5% (248 cases) were major discrepant cases (Class I and II), 34.8% (168 cases) were minor discrepant cases (Cass III and IV) and 11.4% (55 cases) were non-discrepant ones (Class V). The left 11 cases (2.3%) were non-classifiable (Class VI) due to blank clinical diagnosis.

Cases with diagnostic errors (Class I, II, III and IV) accounted for 416 (86.3%) in total. Distribution of cases with diagnostic errors (*n* = 416) were summarized in Table 2. In the major discrepant cases (Class I and II), 64 (25.8%) patients complained of non-indicative symptoms. By contrary, only 18 (8.1%) out of the 223 cases with clinicopathological consistent major diagnosis (Class III, IV and V) were caused by non-indicative complaints (Table 3, *p* = 0.000).

**Table 3:** Correlation of indicative complains with the final outcome of clinicopathological discrepancy^a^

Outcome of discrepancy	Total	Indicative complaints		*P* value
		Yes	No	
Consistency in major diagnosis	223	205	18	0.000
Inconsistency in major diagnosis	248	184	64	

^a^Eleven cases that were non-classifiable (Class VI) were excluded from the total number

**Fig. 2:**
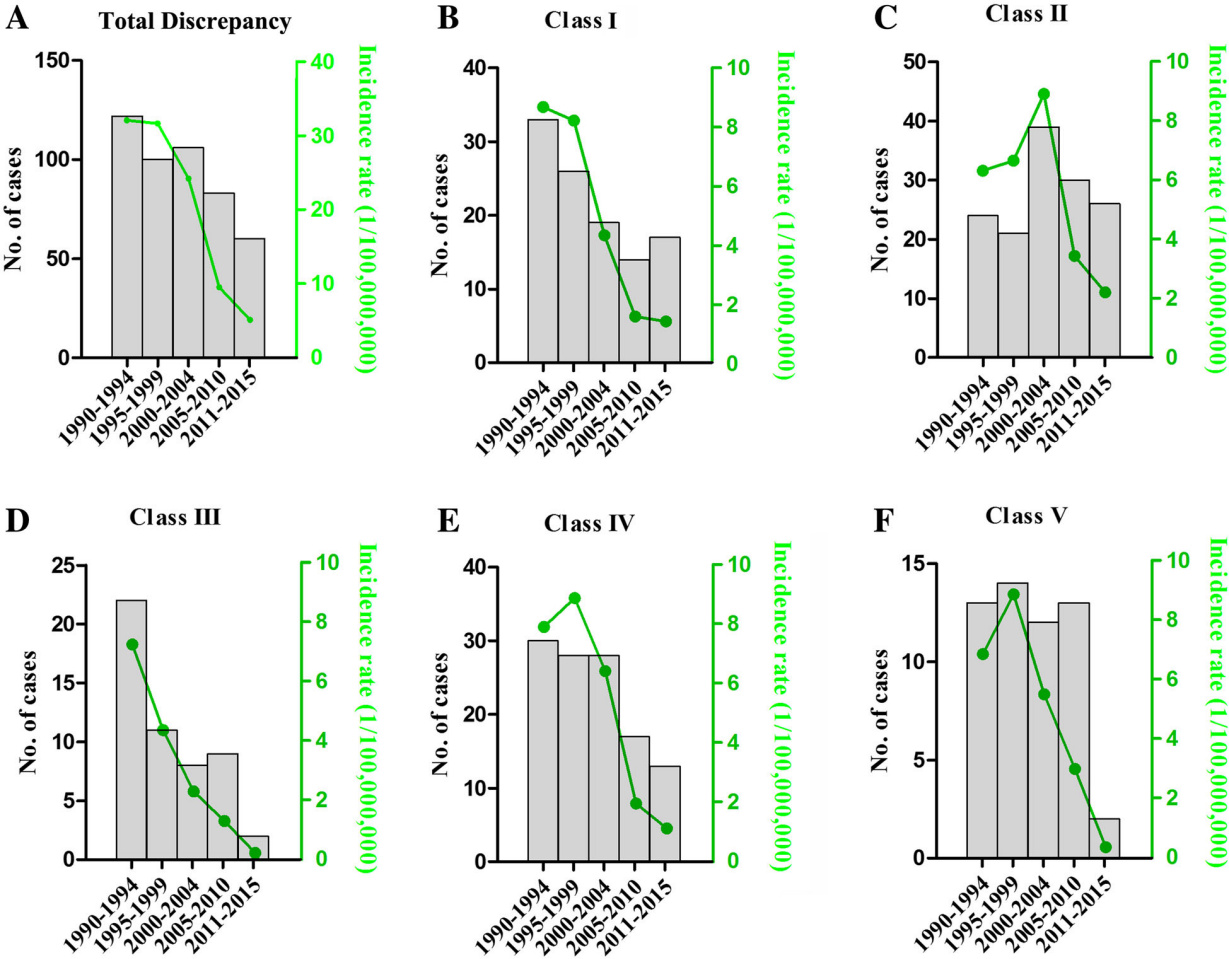
The annual number and incidence rate of cases with diagnostic errors over five periods: 1990–1994, 1995–1999, 2000–2004, 2005–2010 and 2011–2015. **a** The case number and incidence rate of any discrepant cases (Class I to V) were shown and plotted. Total case numbers dropped dramatically (R^2^ = − 0.94, *p* = 0.017) and the incidence rate decreased accordingly (R^2^ = − 0.96, p = 0.01). **b**-**c** The case number and incidence rate of major discrepant cases (Class I and II) were shown. Incidence rate of Class I error steadily dropped (R^2^ = − 0.96, *p* = 0.01), and that of Class II error exhibited an initial increase followed by steady decreases. **d**-**e** The annual number and incidence rate of minor discrepant cases (Class III and IV) were shown. Incidence rate of Class III error steadily dropped (R^2^ = − 0.97, *p* = 0.005), and that of Class IV error exhibited a small increase for the former 10 years but decreased steadily onward (R^2^ = − 0.93, *p* = 0.02). **f** Case numbers of class V discrepancy fluctuated during the period. Its incidence rate exhibited an initial increase, followed by steady decreases onward (R^2^ = − 0.89, *p* = 0.04)

Generally, the total number of discrepant cases (Class I to V) decreased (R^2^ = − 0.94, *p* = 0.017, Fig. 2a) with the incidence rate dropped significantly from the first 5 years to the latest 5 years (R^2^ = − 0.96, *p* = 0.01, Fig. 2a). It had a steady decline in the case numbers of Class I, III and IV discrepancy (Fig. 2b, d and e). Number of class II errors peaked during 2000–2004 and then declined remarkably (Fig. 2c). Incidence rate of Class I error steadily dropped from 8.7 in 1990–1994 to 1.4 per 100, 000, 000 hospitalizations in 2011–2015 (R^2^ = − 0.96, *p* = 0.01, Fig. 2b). Incidence rate of Class II error increased from 6.3 in 1990–1994 years to 8.9 in 2000–2004 years and dropped to 2.2 per 100,000,000 hospitalization in the latest five years (Fig. 2c). Incidence rate of Class III error also steadily dropped from 5.8 in 1990–1994 to only 0.16 per 100,000,000 hospitalizations in 2011–2015 (R^2^ = − 0.97, *p* = 0.005, Fig. 2d). Incidence rate of Class IV error exhibited a small increase for the former 10 years but decreased steadily onward (R^2^ = − 0.93, *p* = 0.02, Fig. 2e). Case numbers of class V errors fluctuated during the period. Its incidence rate exhibited an initial increase, followed by steady decreases onward (R^2^ = − 0.89, *p* = 0.04, Fig. 2f).

Extents of diagnostic concordance strongly associated with organ systems (*p* = 0.0002) and patients’ age (*p* = 0.048) but did not correlate with gender (*p* = 0.73) nor medical settings (*p* = 0.28). There was only a mild correlation between discrepancies and specialties (*p* = 0.09) (Table 4).

**Table 4:** Comparisons between diagnostic errors with patients’ age, gender, medical settings, specialty, and involved systems

Characteristics	I (*n* = 108)	II (*n* = 140)	III (*n* = 53)	IV (*n* = 115)	V (*n* = 55)	*P* value
Age, years						0.048
< 20	17	9	9	11	18	
(20, 40]	19	27	8	27	10	
(40, 60]	37	50	17	34	14	
≥ 60	35	54	19	43	13	
Gender						0.73
Female	39	55	18	50	17	
Male	69	85	35	65	38	
Medical settings						0.28
Tertiary	58	89	32	72	39	
Secondary	42	44	19	36	13	
Primary	8	7	2	7	3	
Specialty						0.09
Internal	40	36	27	44	22	
Surgery	26	50	10	41	16	
Emergency	27	42	10	24	11	
Other wards^a^	15	12	6	6	6	
Organ system						0.0002
Circulatory	20	73	11	28	16	
Respiratory	49	33	20	39	20	
Digestive	31	19	16	28	10	
Other systems^b^	8	15	6	20	9	

^a^includes departments of pediatrics, obstetrics, psychiatry, dermatology, traditional Chinese medicine and EENT

^b^includes central nervous system, urogenital system and endocrine and metabolic system etc

### Predictors of any diagnostic errors, major diagnostic errors and minor diagnostic errors

Since gender and medical setting were not associated with diagnostic discrepancy, these characteristics were not assessed for its predictive values for diagnostic error. Compared with internal medicine (the reference specialty), the odd of the clinical diagnoses being any error (Class I to IV errors) was significantly lower at other wards (i.e. department of pediatrics, or dermatology) (OR = 0.41; 95%CI 0.19–0.86; *p* = 0.02, Table 5). Diseases from the respiratory system had a significantly lower risk of any diagnostic error (OR = 0.48, 95%0.24–0.95, *p* = 0.036). A neoplasm carrier increased by 92% the risk of having any diagnostic error (OR = 1.92; 95%CI 1.18–3.14; *p* = 0.009, Table 5).

**Table 5:** Multivariate predictors of any diagnostic errors (Class I, II, III and IV discrepancies), major errors (Class I and II discrepancies) and minor errors (Class III and IV discrepancies)

Characteristics	Any errors		Major errors		Minor errors	
	Odds ratio (95% CI)	*p* value	Odds ratio (95% CI)	*p* value	Odds ratio (95% CI)	*p* value
Age, years						
(0, 20)	1.14 (0.56–2.32)	0.72	0.52 (0.15–1.82)	0.31	0.35 (0.09–1.36)	0.13
[20, 40)	1.55 (0.88–2.73)	0.12	1.45 (0.58–3.61)	0.42	0.19 (0.36–3.97)	0.77
[40, 60)	1.04 (0.65–1.66)	0.87	0.91 (0.43–1.94)	0.80	0.68 (0.25–1.82)	0.43
≥ 60	1.0 (ref.)		1.0 (ref.)		1.0 (ref.)	
Specialty						
Internal	1.0 (ref.)		1.0 (ref.)		1.0 (ref.)	
Surgery	0.88 (0.51–1.52)	0.65	1.81 (0.71–4.61)	0.21	2.54 (0.85–7.63)	0.09
Emergency	0.77 (0.45–1.30)	0.32	1.58 (0.66–3.78)	0.30	1.48 (0.51–4.35)	0.47
Other wards^a^	0.41 (0.19–0.86)	0.018	1.35 (0.46–3.95)	0.58	0.29 (0.06–1.53)	0.14
Organ system						
Circulatory	0.54 (0.28–1.03)	0.06	0.46 (0.15–1.41)	0.17	0.426 (0.11–1.69)	0.22
Digestive	1.0 (ref.)		1.0 (ref.)		1.0 (ref.)	
Respiratory	0.48 (0.24–0.96)	0.036	0.39 (0.12–1.27)	0.11	0.25 (0.060–0.99)	0.05
Other systems^b^	0.64 (0.33–1.27)	0.20	2.52 (0.77–8.28)	0.12	0.39 (0.09–1.73)	0.21
Length of stay						
≤ 1 day	1.0 (ref.)		1.0 (ref.)		1.0 (ref.)	
(1, 7] days	1.31 (0.76–2.26)	0.32	1.81 (0.74–4.38)	0.19	0.68 (0.22–2.08)	0.50
(7,14] days	1.18 (0.58–2.43)	0.64	1.16 (0.37–3.62)	0.80	0.70 (0.14–3.57)	0.67
> 14 days	1.26 (0.74–2.15)	0.38	1.52 (0.64–3.62)	0.34	0.76 (0.27–2.19)	0.61
Hypertension						
Yes	0.78 (0.42–1.46)	0.44	1.78 (0.64–4.96)	0.27	0.22 (0.06–0.9)	0.036
No	1.0 (ref.)		1.0 (ref.)		1.0 (ref.)	
Neoplasm carrier						
Yes	1.92 (1.18–3.14)	0.009	1.44 (0.63–3.30)	0.39	0.58 (0.23–1.46)	0.25
No	1.0 (ref.)		1.0 (ref.)		1.0 (ref.)	
History of surgery						
Yes	0.98 (0.54–1.78)	0.94	1.53 (0.57–4.09)	0.39	1.68 (0.49–5.75)	0.41
No	1.0 (ref.)		1.0 (ref.)		1.0 (ref.)	
Perioperative period						
Yes	0.96 (0.56–1.66)	0.89	0.74 (0.29–1.84)	0.51	1.65 (0.50–5.40)	0.41
No	1.0 (ref.)		1.0 (ref.)		1.0 (ref.)	

^a^includes departments of pediatrics, obstetrics, psychiatry, dermatology, traditional Chinese medicine and EENT. ^b^includes central nervous system, urogenital system, endocrine and metabolic system etc.

None assessed characteristics were valuable predictors for major diagnostic errors (Class I and II) (Table 5). In the setting of minor errors (Class III and IV) (Table 5), diseases from the respiratory system had a relatively lower risk of minor errors (OR = 0.25, 95%0.06–0.99, *p* = 0.05). A condition with hypertension reduced by 78% the risk of having minor errors (OR = 0.23, 95%CI 0.06–0.91, *p* = 0.036, Table 5).

### Diagnostic discrepancy in primary causes of death

Cardiovascular disease claimed 140 medical disputes, making it the most common cause of death. Of the 140 cardiovascular deaths, 137 (97.8%) had determined extents of discrepancies (Class I to V). Infectious disease claimed 138 disputes, 134 of which had determined extents of discrepancy. Neoplastic disease caused 74 disputes and all of these cases were classifiable of discrepancy (Table 6). The mean length of hospital stay were 15 ± 30 days for cardiovascular disease and 26 ± 72 days for infectious disease. The mean length of stay was 52 ± 92 days for neoplastic disease which was significantly longer than those for cardiovascular disease (*p* = 0.0001) and for infectious disease (*p* = 0.03).

**Table 6:** Associations of diagnostic errors with characteristics in relation to patients’ age, gender, and medical settings in cardiovascular, infectious and neoplastic diseases. Data in each cell was expressed as number of cases

Characteristics	Cardiovascular disease (*n* = 137)						Infectious disease (*n* = 134)						Neoplastic disease (*n* = 74)					
	I	II	III	IV	V	*p* value	I	II	III	IV	V	*p* value	I	II	III	IV	V	*p* value
Age, years						0.01						0.80						0.39
< 40	3	14	4	11	6		17	8	5	9	12		2	3	2	1	2	
[40, 60)	9	32	4	9	3		14	7	4	9	3		1	6	2	8	4	
≥ 60	7	24	2	6	3		11	8	2	15	3		9	10	8	15	1	
Gender						0.92						0.74						0.62
Female	8	28	4	11	5		18	12	1	15	7		2	6	3	11	0	
Male	11	42	6	15	7		27	12	11	18	13		10	13	9	13	7	
Medical settings						0.32						0.78						0.26
Tertiary	10	45	6	17	10		22	16	8	19	11		9	15	8	20	7	
Secondary	9	22	4	9	1		19	7	4	12	8		3	4	4	4	0	
Primary	0	3	0	0	1		4	1	0	2	1		0	0	0	0	0	

For cardiovascular disease, extents of discrepancy significantly associated with patients’ age dying from cardiovascular disease (*p* = 0.01, Table 6). Neither gender nor medical settings was associated with extents of discrepancy in cardiovascular disease (Table 6). No significant correlation was observed between diagnostic discrepancy and characteristics such as patients’ age, gender and medical settings for infectious disease and neoplastic disease (Table 6).

## Discussion

Medical malpractice is a huge problem around the world [1, 2, 11]. Emerging studies have focused on the intensified doctor-patient relationship in China [12]. Several previous studies have also shown the clinic-pathological discrepancy in separate regions in China [3, 13]. All these studies has technically failed to analyze the time trends of medical disputes and diagnostic discrepancy underlying disputed cases over a lengthy period. The present study was the largest autopsy series of medical disputes from China and was the first one to map the scenario of China’s medical disputes to the world elsewhere.

Our data showed that there was a significant year-based decrease in the incidence rate of fatal medical disputes. In parallel, the rates of Class I and III discrepancies declined significantly from 1990 to 2015. Incidence rates of Class II and IV exhibited an initial slight increase around the year 2000 but dropped significantly onwards. The year-based decreases in diagnostic error incidence were basically consistent with two recent studies [8, 14], but in direct contrast to an earlier report which had concluded that the overall major discrepancy rate remained the same since 1960 [7]. Our observation might reflect the rising improvement of diagnostic strategies, especially in the setting of Class I and III discrepant diseases. China has witnessed rapid advances in medical technology and healthcare facilities which largely benefit diagnostic performance for the recent decades [15]. Complicated diseases that would be misdiagnosed in previous years can now be detected at an earlier stage and thereby being intervened prior to progression. On the other hand, stepwise national legislation on medical malpractice may also contribute to the decreased incidence rate of fatal dispute rates. So far, three legislative regulations have been issued on medical disputes in China [4]. The first is the *Rule on the Handling of Medical Accident* established in 1987. The second is the *Regulations on the Handling of Medical Accident* which is effective since 2002 and replaces the previous 1987 regulation [16]. The third regulation is the *Chapter Six Liability for Medical Malpractice of the Tort Law of the People’s Republic of China*, which was adopted by the Standing Committee of the National People’s Congress on December 26, 2009 and became effective in 2010. These stepwise regulations increase the adequacy and fairness of compensation as well as the procedure for resolving medical disputes [4]. The present study observed that the dispute rate remained high before the first round of legislation (mean 0.31 per 1 million patients) but declined dramatically afterwards (R^2^ = − 0.82, *p* < 0.001 for time trends), suggesting that China’s national legislation has successfully achieved its legal effect. However, the decreasing discrepancy rate should not be over-interpreted since not all disputed cases are mandatory to be autopsied in China. Instead, substantial numbers of malpractice claims are addressed through mediation by a third party before they proceeded to authorities for referral to autopsies [3]. Therefore, it is not necessary to conclude that the discrepancy rate keeps going down after each legislation. This conclusion is restricted to only fatal medical disputes which have been referred to autopsy in the certified department.

Despite the year-to-year decreases in diagnostic error, this study still suggests some interesting lessons for healthcare participators.

The present study observed that the vast majority of medical dispute cases were concentrated in tertiary hospitals with the rate of diagnostic errors unfortunately indistinguishable from lower-level medical settings. This is in great difference from other studies which concluded that tertiary teaching hospitals were more likely to associate with fewer diagnostic errors than non-teaching hospitals [10], and death in community hospitals were with an increased chance of discrepancies as compared to university hospitals [17]. The striking finding was also observed in a study by us in Wuhan city, China where tertiary hospitals constituted as high as 59.5% of all medical malpractice cases and comprised 58.8% of diagnostic errors [3]. Reasons for this phenomena might be complex but it should be noted that China has not yet established a mature dividing system for patients. No mature preliminary screening system that filters less complicated cases has been in use. Therefore, patients more likely influx into upper-level healthcare facilities without necessary relation to their disease severity. The current medical system in China might explain the remarkable number of medical disputes and diagnostic errors in upper-level healthcare facilities.

We found that an indicative and accurate complaint on admission positively correlated with the outcome of diagnostic performance, making a direct emphasis on detailed inquiry of patients’ conditions throughout hospitalization. But there were still 82 cases (17%) who made non-indicative complaints, implicating the importance of medical education for patients. In addition, nearly half of cases (47%) died 7 days after hospitalization and 248 patients (51.5%) died of major diagnostic errors. Poor diagnostic performance might be one influential factor for the delayed diagnosis and hence improving diagnostic performance is one thing that could help prevent future misdiagnosis. More importantly, diagnostic negligence should also be claimed. Should there be effective communication between attendant doctors and patients, patients’ survival may be prolonged [18]. Healthcare providers are encouraged to learn lessons from cases of diagnostic errors. It cannot be overemphasized that healthcare providers improve their communication and educational skills [19]. This necessitates timely communication with patients, especially those with undetermined diagnosis after long-term hospitalization, to pay close attention to patients’ dynamic symptoms.

Finally, neoplasm carrier increased by 92% the risk for occurrence of any diagnostic error. This might be due to the fact that neoplasms have less evident symptoms in the early phases. Current diagnostics fail to definitively diagnose early-stage neoplasms [20], leading to easy commitment to major or minor diagnostic error. It is also noteworthy that criteria used in histopathologic diagnosis might be applied in different levels even among experienced pathologists [21]. Therefore, the inter-observer difference in reading of histopathology samples of resected neoplasm might also contribute to the high risk of diagnostic error in neoplastic disease. In addition, the presence of hypertension predict significantly lower risks for occurrence of minor diagnostic errors. This is in great contrast to other studies which concluded the presence of cardiovascular disease increased the chance of discrepancies [10, 22, 23]. Our observation might be due to the fact that hypertension is a chronic progressive disease, and patients with hypertension have regular follow-ups so that attendant physicians know patients’ disease course very well in China. In fact, it has been observed that 39.14% of hypertension patients could control their blood pressure in the normal range, and 34.68% of them could receive more than 4 times follow-ups by the medical technician [24]. Diseases from the respiratory system could also predict a least possibility of any diagnostic errors, which might profit from the improvement of diagnostics such as high-resolution CT [25]. Collectively, these findings might suggest that doctors in China have now been experienced to accurately diagnose hypertension and respiratory diseases.

There are several limitations. First, this study only focused on fatal cases. Non-fatal dispute cases were not referred to the above mentioned department. Hence, the conclusions were restricted to fatal cases with complete autopsy. This study also failed to obtain a nationwide data due to no uniform registry system in China. A multi-institute collaboration containing both fatal and non-fatal cases would aid. Second, the present study only analyzed diagnostic errors underlying medical disputes. The rate of diagnostic errors was significantly higher than previous reports [8, 17, 26]. One explanation was that there was a selection bias since only medically disputed cases were included. Finally, a medical malpractice may be caused by multiple elements such as wrong prescription and suboptimal care, systemic analysis of all contributing elements in addition to diagnostic errors would aid in portraying the whole situation of China’ medical malpractice.

## Conclusion

The discrepancy rate declined over time in the setting of fatal medical dispute cases. Carrying with neoplasm may be a valuable predictor for potential diagnostic errors and diseases from respiratory system and a medical history of hypertension may indicate a least possibility of having minor diagnostic errors.
